# Correlation between adult pyrethroid resistance and knockdown resistance *(kdr)* mutations in *Aedes albopictus* (Diptera: Culicidae) field populations in China

**DOI:** 10.1186/s40249-018-0471-y

**Published:** 2018-09-04

**Authors:** Jing-Peng Gao, Han-Ming Chen, Hua Shi, Heng Peng, Ya-Jun Ma

**Affiliations:** 1 Department of Tropical Infectious Diseases, Second Military Medical University, Shanghai, 200433 China; 2 Institute of Disease Control and Prevention, People’s Liberation Army of China, Beijing, 100071 China; 3 Department of Medical Microbiology and Parasitology, College of Basic Medical Sciences, Second Military Medical University, Shanghai, 200433 China

**Keywords:** *Aedes albopictus*, Pyrethroid, Insecticide resistance, *kdr* mutation, WHO tube bioassay

## Abstract

**Background:**

Arboviral disease transmitted by *Aedes albopictus* such as dengue fever is an important threat to human health. Pyrethroid resistance raises a great challenge for mosquito control. A systematic assessment of *Ae. albopictus* resistance status in China is urgently needed, and the study of correlation between pyrethroid resistance and knockdown resistance (*kdr*) mutations would provide information to guide the control of the *Ae. albopictus* vector.

**Methods:**

Five field populations of *Ae. albopictus* were collected from Jinan (JN), Hangzhou (HZ), Baoshan (BS), Yangpu (YP) and Haikou (HK), China in 2017. Insecticide-impregnated papers were prepared with four pyrethroid chemicals, deltamethrin, permethrin, beta-cypermethrin and lambda-cyhalothrin. The susceptibility of *Ae. albopictus* to pyrethroids was tested by the WHO tube assay. *Kdr* mutations were identified by PCR and sequencing. Moreover, the correlation analysis between *kdr* alleles and pyrethroid resistance was performed.

**Results:**

All five populations of *Ae. albopictus* showed resistance to four pyrethroid insecticides. One *kdr* mutant allele at codon 1532 and three at 1534 were detected with frequency of 5.33% (I1532T), 44.20% (F1534S), 1.83% (F1534 L) and 0.87% (F1534C), respectively. Both 1532 and 1534 mutation mosquitoes were found in the BS and YP populations. Allele I1532T was negatively correlated with deltamethrin resistance phenotype (*OR* < 1), while F1534S mutation was positively correlated with deltamethrin and permethrin resistance (*OR* > 1).

**Conclusions:**

The five field populations of *Ae. albopictus* adults were all resistant to deltamethrin, permethrin, beta-cypermethrin and lambda-cyhalothrin. Mutant F1534S was clearly associated with pyrethroid resistance phenotype in *Ae. albopictus* and this could be developed as a molecular marker to monitor the pyrethroid resistance problem in China.

**Electronic supplementary material:**

The online version of this article (10.1186/s40249-018-0471-y) contains supplementary material, which is available to authorized users.

## Multilingual abstracts

Please see Additional file 1 for translations of the abstract into the five official working languages of the United Nations.

## Background

*Aedes albopictus* Skuse, also called the Asian tiger mosquito, is widely distributed in China [1], and is the primary vector of dengue fever and chikungunya fever in China [2, 3]. The spraying of chemical insecticides to control the vector is one of the most important methods to prevent the arboviral diseases due to absence of effective vaccines and treatments [4, 5]. Pyrethroids have been widely used as indoor/outdoor residual or space sprays for mosquito control in China since the 1980s because of their effectiveness and low toxicity. Especially during the dengue fever outbreaks, pyrethroids were heavily used to control mosquito adults [6, 7]. However, pyrethroid resistance was reported in more and more populations [4, 8], raising a great challenge for mosquito control. The evaluation of chemical susceptibility could provide important information to determine mosquito management strategies. Moreover, understanding the mechanism of mosquito resistance to pyrethroids would be beneficial to development of novel insecticides or application methods.

The mechanisms that mosquitoes have developed for the resistance to pyrethroids include behavioral resistance, target insensitivity and metabolic detoxification [9]. Target insensitivity, also known as knockdown resistance (*kdr*) caused by mutations in the voltage-gated sodium channel (*VGSC*) gene, which is the target site of pyrethroids [10]. Several surveys indicated that pyrethroid resistance in *Culex pipiens pallens* Coquillett, *Anopheles sinensis* Wiedemann and *Ae. aegypti* Linnaeus were associated with *kdr* mutations [11–15]. However, there are few studies about *kdr* mutations in *Ae. albopictus*. In 2011, Kasai et al. [10] firstly reported that the F1534C mutant allele was detected in *Ae. albopictus* from Singapore, with a frequency of 73.1%. Then, in 2015, our research team detected new mutant alleles F1534S and F1534 L in *Ae. albopictus* larvae from Haikou, Hainan, China, and identified that the F1534S mutant allele was associated with pyrethroid resistance [16, 17]. The F1534S/L mutations were also found in *Ae. albopictus* from Guangzhou, Guangdong, China [4, 18].

*Aedes albopictus* has a wide distribution in China, north to Liaoning Province and west to Tibet Autonomous Region [19]. A lot of investigations about the susceptible status of *Ae. albopictus* field populations have been reported in China [16, 20–22], most of which focused on the larvae stage [23]. However, pyrethroid insecticides space spraying was mainly targeted to adult mosquitoes. The susceptible status of the adult stage of *Ae. albopictus* tested by the World Health Organization (WHO) tube assay was limited [7, 18, 24–26]. The diagnostic doses of deltamethrin, permethrin, beta-cypermethrin and lambda-cyhalothrin of *Ae. albopictus* in China were established [27], which could be used as reference for surveillance of physiological resistance. Furthermore, as outbreaks of dengue fever have expanded to new regions in recent years [28–30], a systematic evaluation of *Ae. albopictus* adult resistance status in China is urgently needed.

In this study, we investigated insecticide resistance of *Ae. albopictus* adults collected from five populations in Central, East and South China to four kinds of pyrethroids using the WHO tube assay. The corresponding *kdr* mutations in tested *Ae. albopictus* adults were detected using PCR and sequencing. Moreover, the associations between pyrethroid resistance and *kdr* mutations were analyzed.

## Methods

### Mosquito samples

The field populations of *Ae. albopictus* were collected from five sites in 2017, as Jinan Shandong (JN), Hangzhou Zhejiang (HZ), Baoshan Shanghai (BS), Yangpu Shanghai (YP) and Haikou Hainan (HK), China (Table 1). Larvae and pupae were scooped from breeding sites, such as used tire dumps, flower pot trays, metal containers, terraria, stone holes, ceramic vessels, plastic containers, and other water containers. They were brought back and reared to adults under standard conditions at 26 ± 1 °C and 65 ± 5% relative humidity with a photoperiod of 12-h light: 12-h dark at the insectary of the Second Military Medical University, Shanghai, China. The F_0_ generation adults were used for the susceptibility test.

**Table 1:** The summary information of the *Ae. albopictus* collection sites in China.

Population	Date	Coordinates	Sampling environment	Mosquito-borne diseases^a^
JN	September 2017	117°03′E, 36°39′N	Park, Urban	Malaria [52], Filariasis [53], Japanese B encephalitis [54]
HZ	September 2017	120°08′E, 30°15′N	Park, Suburban	Malaria [52], Dengue fever [8], Filariasis [53], Japanese B encephalitis [54]
BS	September 2017	121°31′E, 31°24′N	Residential area, Urban	Malaria [52], Dengue fever [30], Filariasis [53], Japanese B encephalitis [54]
YP	September 2017	121°33′E, 31°19′N	Park, Suburban	Malaria [52], Filariasis [53], Japanese B encephalitis [54]
HK	August 2017	110°21′E, 20°05′N	Residential area, Urban	Malaria [52], Dengue fever [8], Filariasis [53], Japanese B encephalitis [54]

^a^Local mosquito-borne diseases cases reported in the last 50 years

### Insecticide susceptibility bioassay

*Ae. albopictus* species were identified by morphology [19] and the molecular marker of ITS2 [31]. Female mosquitoes of 3 to 5 days old after emergence and not blood fed were tested for susceptibility to four pyrethroid insecticides by the tube bioassay following the WHO protocol [32]. The insecticide-impregnated test papers of deltamethrin (0.1036%), permethrin (1.0634%), beta-cypermethrin (0.2400%) and lambda-cyhalothrin (0.2372%) were made in our laboratory [27]. The above four technical-grade pyrethroid chemicals were provided by Jiangsu Yangnong Chemical Group Co., Ltd. (deltamethrin, 98.37% purity; lambda-cyhalothrin, 95.85% purity), and Jiangsu Gongcheng Bio-Tech Co., Ltd. (permethrin, 96.20% purity; beta-cypermethrin, 95.00% purity). Silicone oil-treated papers without insecticide were used as a control. Tests with each insecticide paper and untreated paper were repeated for at least 4 times, including approximately 100 female mosquitoes. The number of mosquitoes knocked down was recorded at 1 h. If the mosquitoes lay with ventral side up and could not fly up, they were considered to be knocked down. After 1 h exposure, the mosquitoes were transferred to a recovery tube and maintained on 10% of sucrose solution for 24 h when the number of dead mosquitoes was recorded to calculate mortality rate, which was used to evaluate the insecticide susceptibility status. After 24 h, if the mosquito can fly, it was considered to be alive, regardless of the number of legs remaining; if it was knocked down, whether or not it had lost legs or wings, was considered moribund and was counted as dead. The 24 h mortality was used to calculate the insecticide sensitivity. Dead and surviving mosquitoes were collected and preserved in 95% ethanol for subsequent DNA analysis.

### DNA extraction and *kdr* alleles detection

Genomic DNA was extracted from a single mosquito using DNAzol Reagent (Invitrogen, USA). To identify *kdr* alleles, partial sequences of domains II, III and IV of the *VGSC* gene were amplified using the primers aegSCF3 and aegSCR22, aegSCF7 and albSCR9 (designed in this study, 5’-CTG ATC CTC CGT CAT GAA CA-3′), albSCF6 and albSCR8, developed by Kasai [10]. The PCR kit was purchased from Aidlab, China. PCR reaction was carried out in Veriti 96 well Thermal Cycler (Applied Biosystems, USA). The cycling parameters used were those of Kasai [10]. After electrophoresis, PCR products were purified and directly sequenced in both directions with the same primers.

### Statistical analysis

The mortality data were adjusted according to Abbott’s formula if any mosquitoes in the control tube were dead [33]. The susceptibility status of *Ae. albopictus* was identified by WHO’s criteria, using 90% mortality rate as threshold for resistance [32]. Sequences were aligned and analyzed by DNASTAR Lasergene 12.0 software [34]. The codons were examined and genotypes were determined.

Because none of the populations showed mortality of > 90%, the dead mosquito individuals after the test were considered to have a susceptible phenotype while surviving mosquitoes were categorized as resistant. The *kdr* allele frequencies in resistant and susceptible samples were calculated in each population. Chi-squared tests were used to examine the association between *kdr* alleles and the resistant phenotype. The dependent variables were the mosquito phenotypes (resistant or susceptible) at 24 h after bioassay. Then, the odds ratio (*OR*) values and 95% confidential intervals (*CI*) of *kdr* alleles were calculated using SPSS 20.0 (IBM Corp., Armonk, NY, USA) [35]. The relationship between the *kdr* allele and resistant phenotype was considered as positive when *OR* > 1, while as negative when *OR* < 1. If 95% *CI* of *OR* value was a range across 1 or *P*-value > 0.05, it was considered as statistically insignificant.

## Results

### Pyrethroid susceptibility status of *Ae. albopictus* field populations

**Fig. 1:**
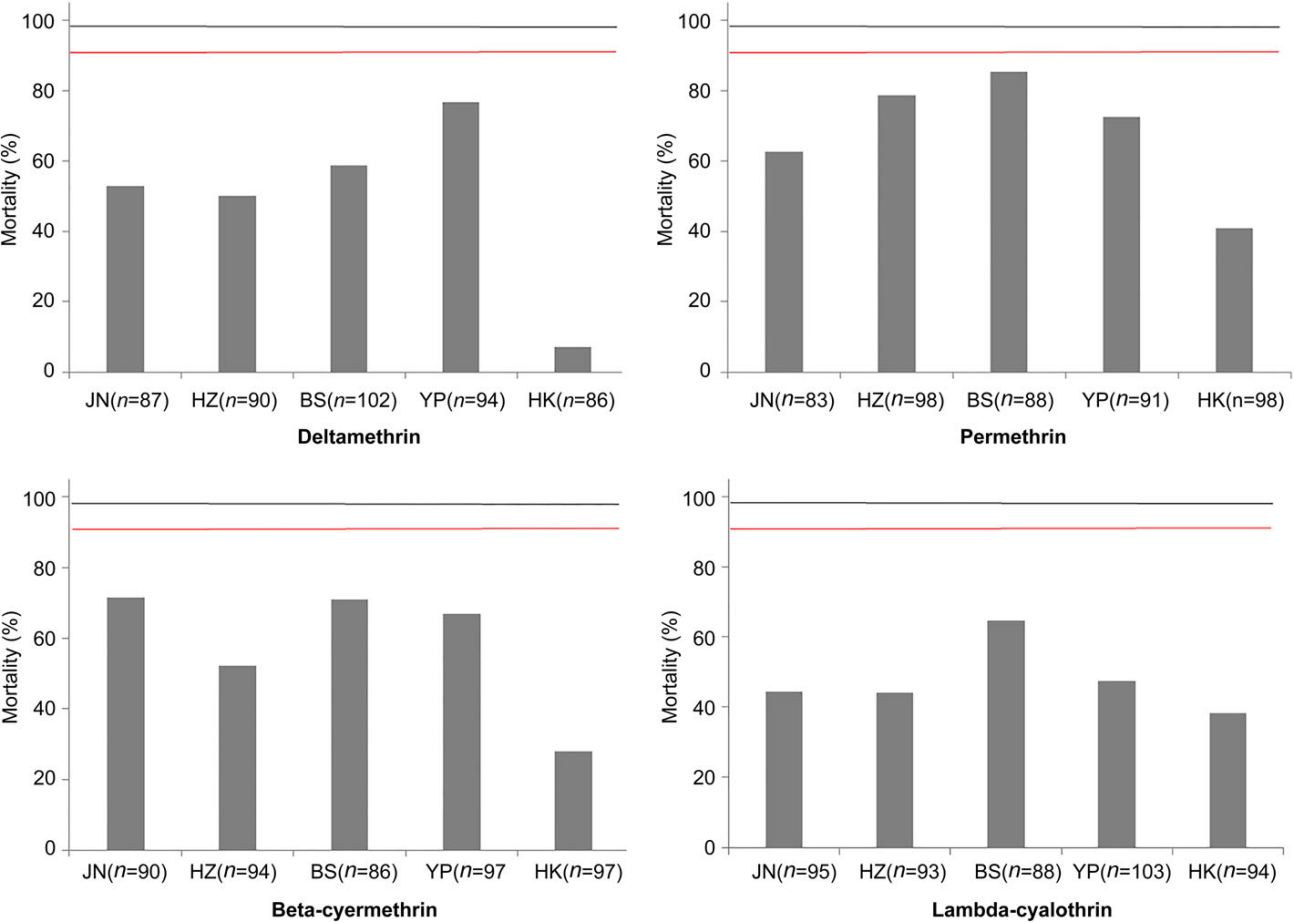
The mortality of *Aedes albopictus* field populations after exposure to four pyrethroids using the WHO tube bioassay. Red lines represent mortality at 90%, black lines as 98%, error bars represent 95% confidential interval (*CI*)

All five populations of *Ae. albopictus* showed resistance to four pyrethroid insecticides (Fig. 1). The range of mortality was 6.98% (HK) − 76.60% (YP) after exposure to deltamethrin, 40.82% (HK) − 85.23% (BS) to permethrin, 27.84% (HK) − 71.58% (JN) to beta-cypermethrin and 38.30% (HK) − 64.77% (BS) to lambda-cyhalothrin (Additional file 2: Table S1). Of the four pyrethroid insecticides tested, the mortality was the lowest after exposure to deltamethrin across all populations. The resistance level of *Ae. albopictus* HK population was all the highest to four pyrethroids tested, while BS or YP samples was relatively low in the five populations.

### *kdr* alleles frequency in *Ae. albopictus* field populations

Sequences of domains II, III and IV of the *VGSC* gene were obtained from 303 mosquitoes exposed to deltamethrin and 326 individuals exposed to permethrin. Non-synonymous *kdr* mutations were detected at codon 1532 and 1534 in domain III of the *VGSC* gene. Synonymous mutations were not recorded in this study, but were found in all three domains.

Two alleles were detected at codon 1532, one mutant allele ACC/T and the wildtype allele ATC/I (GenBank accession No.: MH384955 − MH384958). The allele I1532T was only detected in BS and YP populations with a frequency of 8.03 and 18.00%, respectively. There were three genotypes including the wildtype genotype I/I (75.57%), wildtype/mutant heterozygote I/T (22.90%) and mutant genotype T/T(1.53%) in two populations (Additional file 2: Table S2).

At codon 1534, the wildtype TTC/F frequency was 53.10%, and three *kdr* mutant alleles TCC/S, TTA/L and TGC/C were detected with frequency of 44.20, 1.83 and 0.87%, respectively (GenBank accession No.: MH384950 − MH384961). There were a total of seven genotypes, including wildtype genotype F/F (37.52%), wildtype/mutant heterozygote F/S (28.30%), F/L (2.38%) and F/C (0.48%), and mutant genotype S/S (29.41%), L/L (0.64%) and S/C (1.27%). The mutant allele F1534S was found in all *Ae. albopictus* populations, namely JN, HZ, BS, YP and HK with frequency of 1.39, 90.83, 22.63, 37.20 and 64.75%, respectively, while F1534 L was only found in the JN population with a frequency of 10.65%, and F1534C was only found in the HK population with a frequency of 3.96% (Additional file 2: Table S3).

In the BS and YP populations, we found some individual mosquitoes that showed a *kdr* mutation in both the 1532 and 1534 codons. The genotypes included wildtype+mutant type (I/I + F/S, I/I + S/S, I/T + F/F, T/T + F/F) and both mutant type (I/T + F/S) (Table 2). However, there were no double mutant homozygotes (T/T + S/S) found, which may be due to the low numbers sampled or due to some other reason to do with the genotype itself which causes it to be rare.

**Table 2:** Frequency of simultaneous mutations at codon I1532T and F1534S in *Ae. albopictus* of BS and YP populations from China

Population	*n*	(Wild+mutant) type				(Mutant+mutant) type
		I/I + F/S	I/I + S/S	I/T + F/F	T/T + F/F	I/T + F/S
BS	137	39(28.06)	9(6.47)	18(12.95)	1(0.72)	5(3.60)
YP	125	38(30.40)	18(14.40)	20(16.00)	3(2.40)	19(15.20)

Note: *n* indicates the sample number. Data outside brackets is the number of individuals; data inside brackets means its frequency (%)

### Correlations between pyrethroid resistance and *kdr* mutations

The *OR* values and 95% *CI* of *kdr* mutant alleles at codon 1532 and 1534 in *Ae. albopictus* field populations after exposure to deltamethrin and permethrin were calculated. At codon 1532, *OR* value was 0.49 (*P* < 0.05), and 95% *CI* ranging from 0.23 to 1.00 indicating that I1532T mutant allele was negatively correlated with the deltamethrin resistance phenotype in total samples, and there was no significant correlation in YP and BS populations (Table 3). However, there is no significant correlation between I1532T mutation and permethrin resistant across the total sample, nor in each of the YP and BS populations individually.

**Table 3:** *kdr* mutant allele frequency at codon 1532 of *Ae. albopictus* and association with pyrethroid resistance in BS and YP populations from China

Insecticide	Population	Phenotype	*n*	*kdr* allele		
				I1532T	I1532	*OR* (95% *CI*)
Deltamethrin	BS	R	32	6(9.38)	58(90.63)	2.24 (0.54, 9.37)
		S	34	3(4.41)	65(95.59)	
	YP	R	28	6(10.71)	50(89.29)	0.39 (0.14, 1.05)
		S	38	18(23.68)	58(76.32)	
	Total	R	161	12(3.73)	310(96.27)	0.49^*^ (0.23, 1.00)
		S	142	21(7.39)	263(92.61)	
Permethrin	BS	R	25	4(8.00)	46(92.00)	0.80 (0.23, 2.75)
		S	46	9(9.78)	83(90.22)	
	YP	R	23	7(15.22)	39(84.78)	0.74 (0.28, 2.01)
		S	36	14(19.44)	58(80.56)	
	Total	R	138	11(3.99)	265(96.01)	0.64 (0.31, 1.33)
		S	188	23(6.12)	353(93.88)	

Note: *n* indicates the sample number. S was the susceptible phenotype; R indicates the resistant phenotype. Data outside brackets indicate the number of individuals; data inside brackets indicate frequency (%).

^*^P < 0.05

At codon 1534, F1534S mutant allele was positively correlated with deltamethrin and permethrin resistance in total samples, both *OR* values was above 1 (Table 4). Also, the *OR* values of F1534S mutant allele were all above 1 in three populations (BS, YP and HK) to deltamethrin and two (YP and HK) to permethrin, showed that this allele was positively correlated with the resistance phenotype. Furthermore, there were no statistical significances between F1534 L/F1534C and resistance to two pyrethroids.

**Table 4:** *kdr* mutant allele frequency at codon 1534 of *Ae.albopictus* populations and association with pyrethroid resistance in China

Insecticide	Population	Phenotype	*n*	*kdr* allele				*OR* (95% *CI*)		
				F1534S	F1534 L	F1534C	F1534	F1534S	F1534 L	F1534C
Deltamethrin	JN	R	26	1 (1.92)	6 (11.54)	0 (0.00)	45 (86.54)	2.35 (0.21, 26.73)	1.01 (0.32, 3.22)	–
		S	30	0 (0.00)	7 (11.67)	0 (0.00)	53 (88.33)			
	HZ	R	25	43 (86.00)	0 (0.00)	0 (0.00)	7 (14.00)	0.28 (0.06, 1.42)	–	–
		S	23	44 (95.65)	0 (0.00)	0 (0.00)	2 (4.35)			
	BS	R	32	18 (28.13)	0 (0.00)	0 (0.00)	46 (71.88)	2.94^*^ (1.17, 7.34)	–	–
		S	34	8 (11.76)	0 (0.00)	0 (0.00)	60 (88.24)			
	YP	R	28	30 (53.57)	0 (0.00)	0 (0.00)	26 (46.43)	2.35^*^ (1.16, 4.79)	–	–
		S	38	25 (32.89)	0 (0.00)	0 (0.00)	51 (67.11)			
	HK	R	50	77 (77.00)	0 (0.00)	6 (6.00)	17 (17.00)	7.55^*^ (3.11, 18.34)	–	3.53 (0.63, 19.83)
		S	17	12 (35.29)	0 (0.00)	2 (5.88)	20 (58.82)			
	Total	R	161	169 (52.48)	6 (1.86)	6 (1.86)	141 (43.79)	2.50^*^ (1.79, 3.51)	1.13 (0.37, 3.44)	3.96 (0.79, 19.90)
		S	142	89 (31.34)	7 (2.46)	2 (0.70)	186 (65.49)			
Permethrin	JN	R	20	2 (5.00)	4 (10.00)	0 (0.00)	34 (85.00)	5.06 (0.51, 50.52)	1.14 (0.30, 4.32)	–
		S	32	0 (0.00)	6 (9.38)	0 (0.00)	58 (90.63)			
	HZ	R	34	60 (88.24)	0 (0.00)	0 (0.00)	8 (11.76)	0.53 (0.16, 1.70)	–	–
		S	38	71 (93.42)	0 (0.00)	0 (0.00)	5 (6.580			
	BS	R	25	12 (24.00)	0 (0.00)	0 (0.00)	38 (76.00)	0.90 (0.40, 1.99)	–	–
		S	46	24 (26.09)	0 (0.00)	0 (0.00)	68 (73.91)			
	YP	R	23	20 (43.48)	0 (0.00)	0 (0.00)	26 (56.52)	2.31^*^ (1.05, 5.09)	–	–
		S	36	18 (25.00)	0 (0.00)	0 (0.00)	54 (75.00)			
	HK	R	36	60 (83.33)	0 (0.00)	0 (0.00)	12 (16.67)	6.13^*^ (2.81, 13.38)	–	0.43 (0.05, 3.82)
		S	36	31 (43.06)	0 (0.00)	3 (4.17)	38 (52.78)			
	Total	R	138	154 (55.80)	4 (1.45)	0 (0.00)	118 (42.75)	2.02^*^ (1.47, 2.78)	1.26 (0.35, 4.55)	0.47 (0.05, 4.26)
		S	188	144 (38.30)	6 (1.60)	3 (0.80)	223 (59.31)			

Note: *n* indicates the sample number. S was the susceptible phenotype; R indicates the resistant phenotype. Data outside brackets indicate the number of individuals; data inside brackets indicate frequency (%)

^*^P < 0.05, −, no data

The *kdr* mutant allele F1534S showed a positive correlation with resistance to two pyrethroids, while I1532T as a negative correlation with deltamethrin. It is interesting that the two adjacent mutations have opposite effects on pyrethroid resistance, so we chose the samples with F1534S + I1532T and analyzed the possible interactions using the Chi-squared method. The results showed no statistical significance (Table 5), indicating that there were no associations between I1532T with F1534S with respect to pyrethroid resistance. However, a relatively small sample size may be the cause of insignificant results.

**Table 5:** Association between I1532T and pyrethroid resistance caused by F1534S in *Ae. albopictus* BS and YP populations from China

Insecticide	Population	Phenotype	*n*	I1532T	I1532	*P*-value
Deltamethrin	BS	R	16	2 (6.25)	30 (93.75)	1.000
		S	8	1 (6.25)	15 (93.75)	
	YP	R	24	5 (10.42)	43 (89.58)	0.181
		S	22	9 (20.45)	35 (79.55)	
	Total	R	40	7 (8.75)	73 (91.25)	0.156
		S	30	10 (16.67)	50 (83.33)	
Permethrin	BS	R	9	1 (5.56)	17 (94.44)	0.555
		S	20	1 (2.50)	39 (97.50)	
	YP	R	16	2 (6.25)	30 (93.75)	0.588
		S	15	3 (10.00)	27 (90.00)	
	Total	R	25	3 (6.00)	47 (94.00)	0.948
		S	35	4 (5.71)	66 (94.29)	

Note: All samples have *kdr* mutate allele F1534S. *n* indicates the sample number. Data outside brackets indicate the number of individuals; data inside brackets indicate frequency (%)

## Discussion

In this study, five field populations of *Ae. albopictus* were collected ranging from Central (Jinan Shandong), East (Baoshan Shanghai, Yangpu Shanghai and Hangzhou Zhejiang) and South (Haikou Hainan) China. WHO tube bioassay results showed that all populations were resistant to the four tested pyrethroids. The corresponding *kdr* mutations were detected in the five field populations of *Ae. albopictus*. I1532T (5.33%) at codon 1532 and F1534S (44.20%), F1534 L (1.83%) and F1534C (0.87%) at codon 1534 were found in the tested mosquitoes. Correlative analysis showed that I1532T mutation was negatively correlated with deltamethrin resistance and F1534S mutation was positively correlated with deltamethrin and permethrin resistance phenotype in *Ae. albopictus*.

To explain the resistance of *Ae. albopictus* to pyrethroid insecticides, a survey about the insecticide sales and usage was conducted with the staff of Jiangsu Gongcheng Bio-Tech Co., Ltd., members of the community and pest control organizations in China, and by searching the relevant reports. There were 23 active ingredients of pyrethroids allowed as public health insecticides by the government of the People’s Republic of China [6]. Over the past decade, beta-cypermethrin, lambda-cyhalothrin and permethrin were the most commonly used and marketed pyrethroid insecticides. Deltamethrin was less common because its resistance was reported in many places in China [22, 36–38]. Moreover, according to Wang’s report [6] and “general situation of pesticide production and usage in China in 2015” [39], these four pyrethroids in this testing were heavily used in agriculture, gardens and public health in China. The space spraying frequency was usually once a week in the season of mosquito activity, and even once a day during dengue outbreaks.

In recent years, there have been several outbreaks of dengue fever in China, which have had a tendency to spread to the north [8, 28–30]. As one of the most important control measures, pyrethroid insecticides spraying was widely used in epidemic outbreaks. Few investigations of pyrethroid insecticides resistance of *Ae. albopictus* adult in China have been reported, and those that have been published used a different diagnostic dose from that tested in the current study [18, 26, 40, 41]. Compared to previous reports, we tested the *Ae. albopictus* adult samples from more areas including Central, East and South China. The environment of collecting sites were residential areas or parks, where spraying was applied outside on a regular basis to reduce mosquito adult density, and garden pest control insecticides also aggravated the selection pressure of local mosquitoes. Moreover, the five collecting sites were all located in the mosquito-borne diseases (malaria, filariasis, Japanese B encephalitis) epidemic region in the last 50 years (Table 1), where the usage quantity of chemical insecticides has been high throughout history. Many investigations indicated that *Ae. albopictus* larvae from these areas had developed resistance to pyrethroids [16, 20–22]. *Aedes albopictus* adults from Hangzhou, Zhejiang were all susceptible [26], which was tested by insecticide-impregnated papers with 0.1% deltamethrin, 3% permethrin and 3% beta-cypermethrin. The differences should be further analyzed because there were many factors affecting bioassay results, such as testing samples, breeding sites, and assessment of mortality standard. These results enriched the information on the pyrethroid resistance of *Ae. albopictus* populations from a wider area in China. In addition, pyrethroid resistance was detected in adult mosquitoes, which was more direct evidence to guide the use of insecticides.

Among the five populations, *Ae. albopictus* from Haikou Hainan showed higher resistance level to four pyrethroids. The reason may be that Haikou is in the south of China, located at marginal zone of the tropics where the mosquito population density was high almost all the year. In the past, dengue fever outbreaks have occurred twice in 1979–1982 and 1985–1988 in Hainan Island and surrounding areas; the mortality rate was 0.0785% [42–45]. All the factors led to the continuous use of insecticides and contributed to the development of resistance [16, 17, 37].

The correlation between the *kdr* mutations and pyrethroid resistance was reported in *An. gambiae* [46], *An. sinensis* [15] and *Ae. aegypti* [14]. However, there are few reports about the correlation between the *kdr* mutations and the pyrethroid resistance in despite *kdr* mutant alleles being detected in *Ae. albopictus* field populations [4, 16–18]*.* In order to test for a correlation between *kdr* mutations and pyrethroid resistance of *Ae. albopictus*, we ensured that the mosquitoes tested by the bioassay and the *kdr* gene detection were the same in this study, which was important for determining the association between mutations and pyrethroid resistance. Our results further confirmed that the significant positive correlation between F1534S and pyrethroid resistance phenotype in *Ae. albopictus* (*OR* value was 2.50 and 2.02 for deltamethrin and permethrin), suggesting that F1534S mutant allele is a potential biomarker for surveillance of physiological resistance in China. Similarly, *OR* value of F1534S for deltamethrin was from 9.3 to 33.6 in *Ae. albopictus* Guangzhou populations reported by Li et al. [18]. We are the first to report that the I1532T mutation is negatively correlated with pyrethroid resistance. A similar pattern has been found in which the F1534C mutation in *Ae*. *aegypti* is negatively correlated with pyrethroid resistance in a study from southern China [47]. A different pattern was found from the F1534S mutation in these *Ae. albopictus* samples, I1532T mutation only showed negative correlation with deltamethrin resistance, but no correlation with permethrin. The reason may be that deltamethrin and permethrin are two different types of pyrethroids. Permethrin is a type I pyrethroid, which is without an α-cyano group, while deltamethrin is type II pyrethroid which does contain α-cyano. In this study, F1534 L showed no correlation with pyrethroid resistance (no statistical significance in OR value) (Table 4), which was inconsistent with the results in *Ae. albopictus* Guangdong population, China (range of *OR* value from 15.7 to 19.8) [18]. The correlation between different *kdr* mutant alleles and pyrethroid resistance requires further confirmation in more samples.

The interactions between mutation positions may lead to phenotypic changes in *Ae. aegypti* [48], which is also an interesting issue in mosquito resistance. We found some individuals with both I1532T + F1534S and I1532T + F1534 L mutations in *Ae. albopictus* Shanghai (BS and YP) and Yunnan (JH) population [49]. The occurrence of multiple mutations may be a result of the long-term insecticide pressure on mosquitoes. The analyzed results showed there was no significant correlation between I1532T and pyrethroid resistance caused by F1534S in *Ae. albopictus* BS and YP populations from China. However, a relatively small sample size of the individuals with simultaneous mutations may have led to insignificant results. The interaction between the I1532 with F1534 mutations needs further clarification.

The mechanisms of mosquito insecticide resistance are often multiple [9, 18, 35]. For example, in *An. sinensis*, no *kdr* mutant alleles were found in Yunnan, but metabolic detoxification enzymes (monooxygenases, glutathion S-transferase and carboxylesterases) play major roles in pyrethroids and DDT resistance while *kdr* alleles play a minor role [35]. The *kdr* mutations were reported largely from *An. sinensis* populations in central China, where the resistance to DDT and pyrethroids were conferred primarily by the metabolic detoxification mechanisms as well as the *kdr* mutation [50]. To date, there are few reports of metabolic detoxification mechanisms in *Ae. albopictus* insecticides resistance, and no regular pattern and potential correlations were found [18, 51]. Although we focused on the correlations between *kdr* mutations with the pyrethroid resistance in this research, we believe metabolic detoxification enzymes may also play important roles in pyrethroid resistance in *Ae. albopictus* and will be a topic of future research.

## Conclusions

The field populations of *Ae. albopictus* adults collected from Jinan, Hangzhou, Baoshan, Yangpuand Haikou in China were resistant to deltamethrin, permethrin, beta-cypermethrin and lambda-cyhalothrin tested by WHO tube bioassay. The *kdr* mutation F1534S was positively correlated with deltamethrin and permethrin resistance phenotype, while the I1532T mutation was negatively correlated only with deltamethrin resistance in *Ae. albopictus*. The I1532T + F1534S mutations in the same individual were also detected; the interaction between them needs further study.

## Additional files

Additional file 1: Multilingual abstracts in the five official working languages of the United Nations. (PDF 1038 kb)

Additional file 2: **Table S1.** The knockdown rate and mortality of *Ae. albopictus* field populations exposed to four pyrethroids. **Table S2.** *Kdr* alleles and genotypes at codon 1532 of *Aedes albopictus* field populations. **Table S3.** *Kdr* alleles and genotypes at codon 1534 of *Aedes albopictus* field populations. (DOCX 31 kb)

## Abbreviations

BS: Baoshan
*CI*: Confidence intervals
HK: Haikou
HZ: Hangzhou
JN: Jinan
*kdr*: Knockdown resistance
*OR*: Odds ratio
*VGSC*: Voltage-gated sodium channels
WHO: World Health Organization
YP: Yangpu
