# Supplementary material for: Correlation between adult pyrethroid resistance and knockdown resistance (kdr) mutations in Aedes albopictus (Diptera: Culicidae) field populations in China
Source: Infect Dis Poverty. 2018 Sep 4;7:86. doi: 10.1186/s40249-018-0471-y (PMC6122577; doi:10.1186/s40249-018-0471-y)
Supplement: Supplementary file 1 — Multilingual abstracts in the five official working languages of the United Nations. (PDF 1038 kb) [file 40249_2018_471_MOESM1_ESM.pdf]

Translation of the abstract into the five official working languages of the United Nations

العلاقة المتبادلة بين طفرات مقاومة البيرثرويد البالغة وطفرات المقاومة الحاسمة(***kdr***)في التجمعاتالحقلية للبعوضة الزاعجة المنقطة بالأبيض والتي تنتمي لعائلة (اليرقات: ذوات الجناحين)في الصين

جينغ بينج جاو، هان مينغ تشن، هوا شي، هنغ بنغ و يا جون ما

نبذة

خلفيةالموضوع: تشكل الأمراض الفيروسية المنقولة عبر المفصليات والتي تنقلهاالبعوضة الزاعجة المنقطة بالأبيضمثل حمى الضنك تهديدًا خطيراً لصحة الإنسان. كما تشكل مقاومة البيرثرويد تحديًا هائلًا أمام مكافحة البعوض. لذا هناك احتياج مُلح لإجراء تقييم منهجيلحالة مقاومة البعوضة الزاعجة المنقطة بالأبيضفي الصين، حيث أن دراسة العلاقة المتبادلة بين طفرات مقاومة البيرثرويد وطفرات المقاومة الحاسمة(*kdr*)ستمدنا بالمعلومات اللازمة في إطار مكافحةالبعوضة الزاعجة المنقطة بالأبيضالناقلة للأمراض أيضًا.

الطرق: تم جمع خمس مجموعات حقلية من البعوضة الزاعجة المنقطة بالأبيض من مناطق: جينان ويُرمز لها (JN)، وهانغتشو (HZ)، وباوشان (BS)، ويانغبو(YP)، وهايكو (HK) بالصين في عام 2017. تم إعداد أوراق مُشبعة بمبيدات حشرية مكونة من أربعة مركبات كيميائية لمبيد البيرثرويد هي دلتامثرين، بيرمثرين، بيتا سايبرمثرين، ولامدا سيهالوثرين. تم قياس حساسيةالبعوضة الزاعجة المنقطة بالأبيضللبيرثرويد باستخدام أنبوب اختبار منظمة الصحة العالمية. تم تحديدطفرات المقاومة الحاسمة عن طريق قياس تفاعل البوليميراز المتسلسل واختبار التسلسل. بالإضافة لذلك، تم إجراء تحليل للعلاقة بين أليلات (الصبغة الوراثية متضادة الصفات)المقاومة الحاسمة وبين مقاومة البيرثرويد .

النتائج: أظهرت مجموعات البعوضة الزاعجة المنقطة بالأبيض الخمس كاملةًمقاومة لمركبات البيرثرويد الأربعة. تم اكتشاف طفرة جينية متضادة الصفات(أليل) ضمن المقاومة الحاسمة في الكودون 1532 وثلاثة في الكودون 1534 بتتابع نسبته 5.33% (I1532T)، و 44.20% (F1534S)، و 1.83% (F1534L)، و 0.87% (F1534C) تباعًا. تم العثور على كلٍ من الطفرات الجينية 1532 و 1534 في البعوضيات المُجمعة من منطقتي باوشان (BS) ويانغبو (YP). ارتبطت الطفرة الجينية متضادة الصفات (الأليل) I1532T سلبيًا مع النمط الظاهري لمقاومة مركب دلتامثرين بمعدل يساوي 1 أو أقل ($1 > OR$)، في حين ارتبطت الطفرة الجينية F1534S مع مقاومة مركبي دلتامثرين وبيرميثرين إيجابيًا بمعدل يساوي 1 أو أكثر ($1 < OR$).

الخلاصة: أظهرت مجموعات البعوضة الزاعجة المنقطة بالأبيض البالغة الخمس مقاومة لمركبات دلتامثرين، بيرميثرين، بيتا سايبرمثرين ولامدا سيهالوثرين. ارتبطت الطفرة الجينية F1534S بالنمط الظاهري لمقاومة البعوضة الزاعجة المنقطة بالأبيضللبيرثرويد ارتباطًا جليًا بحيث يمكن تطوير هذا ليصبح مؤشرًا جزيئيًا لرصد مشكلة مقاومة البيريثرويد في الصين.

Translated from English version into Arabic by Eman Shahen, proofread by Heba Kandel, through

中国白纹伊蚊（双翅目：蚊科）现场种群击倒抗性基因突变与拟除虫菊酯类杀虫剂抗性的相关性研究

高景鹏，陈翰明，石华，彭恒，马雅军

摘要

引言：由白纹伊蚊传播的虫媒病如登革热严重威胁人类健康。拟除虫菊酯类杀虫剂（菊酯类）抗性的出现对蚊虫防控提出了巨大的挑战。对中国当前白纹伊蚊现场种群的杀虫剂抗性状况进行系统评估十分必要，并且研究菊酯类抗性与击倒抗性基因（*kdr*）突变的相关性将为白纹伊蚊的防控提供科学依据。

方法：于 2017 年在中国的山东济南（JN）、浙江杭州（HZ）、上海宝山（BS）、上海杨浦（YP）和海南海口（HK）等 5 个地区采集白纹伊蚊的现场种群。制备溴氰菊酯、氯菊酯、高效氯氰菊酯和高效氯氟氰菊酯 4 种药

膜，使用世界卫生组织的接触筒法测定白纹伊蚊成虫对菊酯类杀虫剂的敏感性。测序确定测试样本的 *kdr* 基因突变情况，对应分析了 *kdr* 等位基因与菊酯类杀虫剂抗性的相关性。
结果：中国 5 个白纹伊蚊现场种群对 4 种菊酯类杀虫剂均已产生抗性，检测到 *kdr* 基因第 3 编码区的 1532 位点的 1 个突变等位基因和 1534 位点的 3 个突变等位基因，突变频率分别为 5.33%（I1532T），44.20%（F1534S），1.83%（F1534L）和 0.87%（F1534C）。在 YP 和 BS 种群中，存在 1532 和 1534 两个位点同时突变的情况。I1532T 与溴氰菊酯抗性表型呈负相关关系（*OR*＜1），而 F1534S 突变与溴氰菊酯和氯菊酯抗性呈正相关关系（*OR*＞1）。
结论：中国白纹伊蚊 5 个现场种群对溴氰菊酯、氯菊酯、高效氯氰菊酯和高效氟氯氰菊酯均已产生抗性。*kdr* 突变等位基因 F1534S 与白纹伊蚊菊酯抗性表型存在明显的相关性，可作为中国白纹伊蚊对菊酯类杀虫剂抗性监测的分子标志。

Translated from English version into Chinese by Jing-Peng Gao

Correlación entre las mutaciones de la resistencia a piretroides en adultos y la resistencia knockdown *(kdr)* o resistencia de derribo en poblaciones rurales al*Aedes albopictus* (Diptera: Culicidae) en China

Jing-Peng Gao, Han-Ming Chen, Hua Shi, Heng Peng y Ya-Jun Ma

Resumen
Antecedentes: La enfermedad arboviral transmitida por el *Aedes albopictus* como la fiebre del dengue es una gran amenaza para la salud. La resistencia a piretroides se trata de un gran reto para el control de los mosquitos. Se necesita con urgencia una valoración sistemática del estado de la resistencia al *Ae. albopictus* en China, y el estudio de la correlación entre las mutaciones de la resistencia de los piretroides en adultos y la resistencia al choque (*kdr*) proporcionaría información para guiar el control del vector *Ae. albopictus*.
Métodos: Se recogieron cinco poblaciones campesinas de *Ae. albopictus* en Jinan (JN), Hangzhou (HZ), Baoshan (BS), Yangpu (YP) y Haikou (HK), China en 2017. Se prepararon papeles impregnados con insecticida con cuatro productos químicos piretroides, deltametrina, permetrina, beta-cipermetrina y lambda-cihalotrina. Se comprobó la susceptibilidad del *Ae. albopictus* a los piretroidos mediante el análisis de la OMS. Se identificaron mutaciones *Kdr* por PCR y secuenciación. Además, se realizó el análisis de correlación entre alelos*kdr* y la resistencia a los piretroides.
Resultados: Las cinco poblaciones de *Ae. albopictus* mostraron resistencia a cuatro insecticidas piretroides. Se detectó un alelo mutante*kdr* en el codón 1532 y tres en el 1534 con una frecuencia de 5,33% (I1532T), 44,20% (F1534S), 1,83% (F1534L) y 0,87% (F1534C), respectivamente. Se encontraron mosquitos con mutaciones 1532 y 1534 en las poblaciones BS y YP. El alelo I1532T se correlacionó negativamente con el fenotipo de resistencia a la deltametrina ($O < 1$), mientras que la mutación F1534S se correlación positivamente con la resistencia a la deltametrina y a la permetrina ($O > 1$).
Conclusiones: Las cinco poblaciones de *Ae. albopictus* adultos mostraron resistencia a la deltametrina, permetrina, beta-cipermetrina y lambda-cihalotrina. El mutante F1534S se asoció claramente con el fenotipo de resistencia a piretroides en *Ae. albopictus* y podría desarrollarse como marcador molecular para monitorizar el problema de la resistencia piretroide en China.

Translated from English version into French by Patricia Barthélémy, proofread by Sophie N, through

TRANSLATORS
WITHOUT BORDERS

Корреляция между мутациями взрослых особей с резистентностью к пиретроидам и резистентностью к нокдаун-эффекту *(kdr)* в полевых популяциях *Aedes albopictus* (двукрылые: настоящие комары) в Китае

Цзин-Пэн Гао, Хань-Мин Чэнь, Хуа Ши, Хэн Пэн и Я-Цзюнь Ма

Аннотация

Справочная информация: Арбовирусные заболевания, переносимые *Aedes albopictus,* такие как лихорадка денге, представляют собой серьёзную угрозу для здоровья человека. Резистентность к пиретроидам представляет собой большую проблему в борьбе с комарами. Существует острая необходимость систематической оценки статуса резистентности *Ae. albopictus* в Китае, тогда как исследование корреляции между мутациями как с резистентностью к пиретроидам, так и с резистентностью к нокдаун-эффекту (*kdr*) способно предоставить информацию для борьбы с переносчиками *Ae. albopictus*.

Методы: В 2017 году в провинциях Китая Цзинань, Ханчжоу, Баошань, Янпу и Хайкоу были собраны пять полевых популяций *Ae. albopictus.* Были подготовлены пропитанные инсектицидом куски бумаги с использованием четырёх пиретроидных химических веществ: дельтаметрина, перметрина, бета-циперметрина и лямбда-цигалотрина. Тестирование восприимчивости *Ae. albopictus* к пиретроидам производилось с помощью анализа в пробирках по методу ВОЗ. Выявление мутаций*kdr* осуществлялось с помощью анализа ПЦР и секвенирования. Кроме того, был проведен анализ корреляций между аллелями *kdr* и резистентностью к пиретроидам.

Результаты: Все пять популяций *Ae. albopictus* показали резистентность к четырём пиретроидным инсектицидам. Одна аллель с мутацией *kdr* в кодоне 1532 и три аллели с этой мутацией в кодоне 1534 выявлялись с частотой 5,33% (I1532T); 44,20% (F1534S); 1,83% (F1534L) и 0,87% (F1534C), соответственно. В популяциях провинций Баошань и Янпу были обнаружены обе мутации комаров: 1532 и 1534. Аллель I1532T отрицательно коррелировала с фенотипом резистентности к дельтаметрину ($OR < 1$), в то время как мутация F1534S положительно коррелировала с резистентностью к дельтаметрину и перметрину ($OR > 1$).

Выводы: Все взрослые особи из пяти полевых популяций *Ae. albopictus* были устойчивы к дельтаметрину, перметрину, бета-циперметрину и лямбда-цигалотрину. Мутация F1534S чётко ассоциировалась с фенотипом резистентности к пиретроидам в популяции *Ae. albopictus*, и эту взаимосвязь можно использовать для разработки молекулярного маркера с целью мониторинга проблемы резистентности к пиретроидам в Китае.

Translated from English version into Russian by Dream Translator, proofread by Tatiana Kary, through

TRANSLATORS
WITHOUT BORDERS

Correlación entre las mutaciones de la resistencia a piretroides en adultos y la resistencia knockdown *(kdr)* o resistencia de derribo en poblaciones rurales al*Aedes albopictus* (Diptera: Culicidae) en China

Jing-Peng Gao, Han-Ming Chen, Hua Shi, Heng Peng y Ya-Jun Ma

Resumen

Antecedentes: La enfermedad arboviral transmitida por el *Aedes albopictus* como la fiebre del denge es una gran amenaza para la salud. La resistencia a piretroides se trata de un gran reto para el control de los mosquitos. Se necesita con urgencia una valoración sistemática del estado de la resistencia al *Ae. albopictus* en China, y el estudio de la correlación entre las mutaciones de la resistencia de los piretroides en adultos y la resistencia al choque (*kdr*) proporcionaría información para guiar el control del vector *Ae. albopictus*.

Métodos: Se recogieron cinco poblaciones campesinas de *Ae. albopictus* en Jinan (JN), Hangzhou (HZ), Baoshan (BS), Yangpu (YP) y Haikou (HK), China en 2017. Se prepararon papeles impregnados con insecticida con cuatro productos químicos piretroides, deltametrina, permetrina, beta-cipermetrina y lambda-cihalotrina. Se comprobó la susceptibilidad del *Ae. albopictus* a los piretroidos mediante el análisis de la OMS. Se identificaron mutaciones *Kdr* por PCR y secuenciación. Además, se realizó el análisis de correlación entre alelos*kdr* y la resistencia a los piretroides.

Resultados: Las cinco poblaciones de *Ae. albopictus* mostraron resistencia a cuatro insecticidas piretroides. Se detectó un alelo mutante*kdr* en el codón 1532 y tres en el 1534 con una frecuencia de 5,33% (I1532T), 44,20% (F1534S), 1,83% (F1534L) y 0,87% (F1534C), respectivamente. Se encontraron mosquitos con mutaciones 1532 y 1534 en las poblaciones BS y YP. El alelo I1532T se correlacionó negativamente con el fenotipo de resistencia a la deltametrina ($O < 1$), mientras que la mutación F1534S se correlación positivamente con la resistencia a la deltametrina y a la permetrina ($O > 1$).

Conclusiones: Las cinco poblaciones de *Ae. albopictus* adultos mostraron resistencia a la deltametrina, permetrina, beta-cipermetrina y lambda-cihalotrina. El mutante F1534S se asoció claramente con el fenotipo de resistencia a piretroides en *Ae. albopictus* y podría desarrollarse como marcador molecular para monitorizar el problema de la resistencia piretroide en China.

Translated from English version into Spanish by Fran Egea, proofread by Maria Alejandra Aguada, through
