# Supplementary material for: Correlation between adult pyrethroid resistance and knockdown resistance (kdr) mutations in Aedes albopictus (Diptera: Culicidae) field populations in China
Source: Infect Dis Poverty. 2018 Sep 4;7:86. doi: 10.1186/s40249-018-0471-y (PMC6122577; doi:10.1186/s40249-018-0471-y)
Supplement: Supplementary file 2 — Table S1. The knockdown rate and mortality of Ae. albopictus field populations exposed to four pyrethroids. Table S2. Kdr alleles and genotypes at codon 1532 of Aedes albopictus field populations. Table S3. Kdr alleles and genotypes at codon 1534 of Aedes albopictus field populations. (DOCX 31 kb) [file 40249_2018_471_MOESM2_ESM.docx]

Table S1. The knockdown rate and mortality of *Ae. albopictus* field populations exposed to four pyrethroids.

| Insecticide | Population | N | Knockdown  number(N) | Knockdown  rate(%) | Dead  number(N) | Mortality(%) |
| --- | --- | --- | --- | --- | --- | --- |
| Deltamethrin | JN | 87 | 87 | 100.00 | 46 | 52.87 |
|  | HZ | 90 | 90 | 100.00 | 45 | 50.00 |
|  | BS | 102 | 102 | 100.00 | 60 | 58.82 |
|  | YP | 94 | 94 | 100.00 | 72 | 76.60 |
|  | HK | 86 | 62 | 72.09 | 6 | 6.98 |
| Permethrin | JN | 83 | 61 | 73.49 | 52 | 62.65 |
|  | HZ | 98 | 70 | 71.43 | 77 | 78.57 |
|  | BS | 88 | 80 | 90.91 | 75 | 85.23 |
|  | YP | 91 | 65 | 71.43 | 66 | 72.53 |
|  | HK | 98 | 42 | 42.86 | 40 | 40.82 |
| Beta-  cypermethrin | JN | 90 | 90 | 100.00 | 68 | 71.58 |
|  | HZ | 94 | 94 | 100.00 | 49 | 52.13 |
|  | BS | 86 | 86 | 100.00 | 61 | 70.93 |
|  | YP | 97 | 97 | 100.00 | 65 | 67.01 |
|  | HK | 97 | 46 | 47.42 | 27 | 27.84 |
| Lambda-  cyhalothrin | JN | 95 | 95 | 100.00 | 40 | 44.44 |
|  | HZ | 93 | 93 | 100.00 | 41 | 44.09 |
|  | BS | 88 | 88 | 100.00 | 57 | 64.77 |
|  | YP | 103 | 103 | 100.00 | 49 | 47.57 |
|  | HK | 94 | 36 | 38.30 | 36 | 38.30 |

Note: N indicates the sample number.

Table S2. *Kdr* alleles and genotypes at codon 1532 of *Aedes albopictus* field populations.

| Population | N | *Kdr* allele | |  | *Kdr* genotypes | | |
| --- | --- | --- | --- | --- | --- | --- | --- |
|  |  | Wildtype  ATC/I | Mutant  ACC/T |  | Wildtype  I/I | Wildtype/Mutant  I/T | Mutant  T/T |
| BS | 137 | 252(91.97) | 22(8.03) |  | 115(83.94) | 21(15.33) | 1 (0.73) |
| YP | 125 | 205(82.00) | 45(18.00) |  | 83(66.40) | 39(31.20) | 3 (2.40) |
| Total | 262 | 457(87.21) | 67(12.79) |  | 198(75.57) | 60(22.90) | 4(1.53) |

Note: N indicates the sample number. Data outside brackets was the number of individuals; data inside brackets means frequency (%).

Table S3. *Kdr* alleles and genotypes at codon 1534 of *Aedes albopictus* field populations.

| Population | N | *Kdr* allele | | | | |  | *Kdr* genotypes | | | | | | | | |
| --- | --- | --- | --- | --- | --- | --- | --- | --- | --- | --- | --- | --- | --- | --- | --- | --- |
|  |  | Wildtype |  | Mutant | | |  | Wildtype |  | Wildtype/ Mutant | | |  | Mutant | | |
|  |  | TTC/F |  | TCC/S | TTA/L | TGC/C |  | F/F |  | F/S | F/L | F/C |  | S/S | L/L | S/C |
| JN | 108 | 190(87.96) |  | 3(1.39) | 23(10.65) | 0(0.00) |  | 86(79.63) |  | 3(2.78) | 15(13.89) | 0(0.00) |  | 0(0.00) | 4(3.70) | 0(0.00) |
| HZ | 120 | 22(9.17) |  | 218(90.83) | 0(0.00) | 0(0.00) |  | 2(1.67) |  | 18(15.00) | 0(0.00) | 0(0.00) |  | 100(83.33) | 0(0.00) | 0(0.00) |
| BS | 137 | 212(77.37) |  | 62(22.63) | 0(0.00) | 0(0.00) |  | 84(61.31) |  | 44(32.12) | 0(0.00) | 0(0.00) |  | 9(6.57) | 0(0.00) | 0(0.00) |
| YP | 125 | 157(62.80) |  | 93(37.20) | 0(0.00) | 0(0.00) |  | 50(40.00) |  | 57(45.60) | 0(0.00) | 0(0.00) |  | 18(14.40) | 0(0.00) | 0(0.00) |
| HK | 139 | 87(31.29) |  | 180(64.75) | 0(0.00) | 11(3.96) |  | 14(10.07) |  | 56(40.29) | 0(0.00) | 3(2.16) |  | 58(41.73) | 0(0.00) | 8(5.76) |
| Total | 629 | 668(53.10) |  | 556(44.20) | 23(1.83) | 11(0.87) |  | 236(37.52) |  | 178(28.30) | 15(2.38) | 3(0.48) |  | 185(29.41) | 4(0.64) | 8(1.27) |

Note: N indicates the sample number. Data outside brackets was the number of individuals; data inside brackets means frequency (%).
